# Genetic diagnosis of developmental disorders in the DDD study: a scalable analysis of genome-wide research data

**DOI:** 10.1016/S0140-6736(14)61705-0

**Published:** 2015-04-04

**Authors:** Caroline F Wright, Tomas W Fitzgerald, Wendy D Jones, Stephen Clayton, Jeremy F McRae, Margriet van Kogelenberg, Daniel A King, Kirsty Ambridge, Daniel M Barrett, Tanya Bayzetinova, A Paul Bevan, Eugene Bragin, Eleni A Chatzimichali, Susan Gribble, Philip Jones, Netravathi Krishnappa, Laura E Mason, Ray Miller, Katherine I Morley, Vijaya Parthiban, Elena Prigmore, Diana Rajan, Alejandro Sifrim, G Jawahar Swaminathan, Adrian R Tivey, Anna Middleton, Michael Parker, Nigel P Carter, Jeffrey C Barrett, Matthew E Hurles, David R FitzPatrick, Helen V Firth, on behalf of the DDD study

**Affiliations:** a Wellcome Trust Sanger Institute, Wellcome Trust Genome Campus, Cambridge, UK; b Institute of Psychiatry, King's College London, London, UK; c Melbourne School of Population and Global Health, The University of Melbourne, Melbourne, VIC, Australia; d The Ethox Centre, Nuffield Department of Population Health University of Oxford, Old Road Campus, Oxford, UK; e MRC Human Genetics Unit, MRC IGMM, University of Edinburgh, WGH, Edinburgh, UK; f Cambridge University Hospitals Foundation Trust, Addenbrooke's Hospital, Cambridge, UK

## Abstract

**Background:**

Human genome sequencing has transformed our understanding of genomic variation and its relevance to health and disease, and is now starting to enter clinical practice for the diagnosis of rare diseases. The question of whether and how some categories of genomic findings should be shared with individual research participants is currently a topic of international debate, and development of robust analytical workflows to identify and communicate clinically relevant variants is paramount.

**Methods:**

The Deciphering Developmental Disorders (DDD) study has developed a UK-wide patient recruitment network involving over 180 clinicians across all 24 regional genetics services, and has performed genome-wide microarray and whole exome sequencing on children with undiagnosed developmental disorders and their parents. After data analysis, pertinent genomic variants were returned to individual research participants via their local clinical genetics team.

**Findings:**

Around 80 000 genomic variants were identified from exome sequencing and microarray analysis in each individual, of which on average 400 were rare and predicted to be protein altering. By focusing only on de novo and segregating variants in known developmental disorder genes, we achieved a diagnostic yield of 27% among 1133 previously investigated yet undiagnosed children with developmental disorders, whilst minimising incidental findings. In families with developmentally normal parents, whole exome sequencing of the child and both parents resulted in a 10-fold reduction in the number of potential causal variants that needed clinical evaluation compared to sequencing only the child. Most diagnostic variants identified in known genes were novel and not present in current databases of known disease variation.

**Interpretation:**

Implementation of a robust translational genomics workflow is achievable within a large-scale rare disease research study to allow feedback of potentially diagnostic findings to clinicians and research participants. Systematic recording of relevant clinical data, curation of a gene–phenotype knowledge base, and development of clinical decision support software are needed in addition to automated exclusion of almost all variants, which is crucial for scalable prioritisation and review of possible diagnostic variants. However, the resource requirements of development and maintenance of a clinical reporting system within a research setting are substantial.

**Funding:**

Health Innovation Challenge Fund, a parallel funding partnership between the Wellcome Trust and the UK Department of Health.

## Introduction

The increasing use of whole exome and whole genome sequencing in both research^1^, ^2^ and clinical practice^3^, ^4^, ^5^ raises questions about how to maximise the diagnostic usefulness of genomic data and how to share results with research participants and patients. Furthermore, it is increasingly deemed ethically desirable to return clinically useful results to research participants.^6^ However, return of individual genomic results poses major logistical challenges. A robust workflow must be developed to track individual samples and datasets, generate high-quality genomic data, filter out a very large number of probably benign variants, prioritise plausibly pathogenic variants, and link these findings to individual clinical data for interpretation and appropriate clinical follow-up. Health-related findings from a human genome could potentially include thousands of variants pertaining to hundreds of different conditions,^7^ almost none of which provide clinically useful information for a specific individual.^8^ A first step toward addressing these challenges is to separate potential genomic findings into those that are pertinent to a particular disease investigation and those that are non-pertinent (or incidental) to that disease. Although many commentators have debated the merits of returning different classes of findings from large research studies and biobanks,^6^, ^9^, ^10^ none has yet provided a scalable implementation from patient identification through to a confirmed diagnosis within a research context. Here we describe the development and implementation of a translational genomics workflow in a large-scale rare disease research study to communicate pertinent findings to individual research participants, whilst minimising incidental findings.

The Deciphering Developmental Disorders (DDD) study^11^ is a UK-wide collaborative project that seeks to facilitate the translation of genomic sequencing technologies into the National Health Service (NHS), by collecting a set of high-resolution genomic and phenotypic data for children with severe undiagnosed developmental disorders and their parents. Although whole genome microarray analysis has already proven invaluable for identification of large pathogenic copy number variants (mostly deletions and duplications) in children with developmental disorders,^12^ most children remain undiagnosed. In many cases, the condition is caused by a de novo mutation that occurs spontaneously in the affected child somewhere in the genome,^13^, ^14^, ^15^ and if there is no family history of the condition the genetic basis of the diagnosis can be easily overlooked. The recruitment criteria for the study are focused on congenital or early onset severe phenotypes, and were specifically designed to maximise the chance of finding a highly penetrant monogenic cause for the child's condition. The study was established with the dual aim of assisting the translation of new high-throughput genomic technologies into clinical practice, and elucidating the underlying genetic architecture of developmental disorders. Cambridge South Research Ethics Committee (REC) approved the feedback of potentially causal variants from DDD to the patients' regional genetics centre, whose responsibility it is to assess and validate the findings before communicating them to the families with appropriate counselling regarding recurrence risk, likely prognosis, and potential clinical management. The judgment not to feed back incidental findings was REC-approved, and the protocol and patient information sheets clearly state that “incidental findings will not be reported back in the DDD study”. Nonetheless, we developed a parallel research study to investigate attitudes towards feeding back a broader range of genomic results to research participants.^16^, ^17^

We outline a process to identify and report likely causal variants (pertinent findings) in individual patients, and summarise the results to date. There are many different classes of disease-causing genetic variation, of which some are observed infrequently (eg, uniparental disomy, in which both copies of a single chromosome are inherited from one parent, which occurs in fewer than one in 1000 individuals^18^), whereas others are observed in huge numbers (eg, single DNA base changes, of which every individual has millions in their genome). Different genetic changes need different analysis methods; in particular, more numerous forms of genetic variation need a scalable automated approach. We describe the workflow we have developed to achieve a scalable genome-wide diagnostic analysis, focusing particularly on the automated part of a larger workflow, and we show the clinical usefulness of this workflow using data for 1133 probands with severe undiagnosed developmental disorders as an example.

## Methods

### Clinical data collection

**Figure 1:**
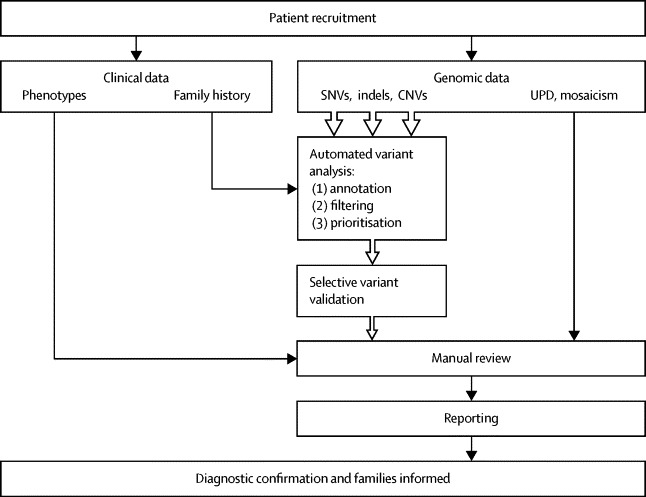
Study workflow SNV=single nucleotide variant. Indel=insertion or deletion. CNV=copy number variant. UPD=uniparental disomy.

We developed a workflow to facilitate patient recruitment, sample tracking, data generation, data analysis, variant filtering, manual curation, and feedback of results (figure 1; appendix 1). Clinically ascertained undiagnosed patients meeting the recruitment criteria (severe undiagnosed neurodevelopmental disorder and/or congenital anomalies, abnormal growth parameters, dysmorphic features, and unusual behavioural phenotypes) were recruited to the DDD study by their UK NHS or Irish Regional Genetics Service, who also recorded clinical information and phenotypes using the Human Phenotype Ontology (HPO)^19^ via a secure web portal within the DECIPHER database.^20^ A team of research coordinators (typically research nurses and genetic counsellors) working in each of the regional services provided essential support with informed consent, sample collection, and data entry (antenatal and growth data, developmental milestones, family history, previous genetic testing, etc) with the patient's phenotype being entered by their clinical geneticist. The study has UK Research Ethics Committee approval (10/H0305/83, granted by the Cambridge South REC, and GEN/284/12 granted by the Republic of Ireland REC).

### Genomic assays

Saliva samples from patients and their parents were collected (Oragene DNA collection kits, DNA Genotek, Kanata, ON, Canada) and DNA extracted (QIAsymphony, Qiagen, Venlo, Netherlands); blood-derived DNA from the child was also provided by the regional genetics laboratories. DNA samples from patients and their parents were analysed at the Wellcome Trust Sanger Institute with microarray analysis (Agilent 2x1M array CGH [Santa Clara, CA, USA] and Illumina 800K SNP genotyping [San Diego, CA, USA]) to identify copy number variants (CNVs) in the child, and exome sequencing (Agilent SureSelect 55MB Exome Plus with Illumina HiSeq) to investigate single nucleotide variants (SNVs), small insertion-deletions (indels), and CNVs in coding regions of the genome (appendix 1). Putative de novo sequence variants identified using DeNovoGear^21^ were validated with targeted Sanger sequencing. The population prevalence (minor allele frequency) of each variant in nearly 15 000 samples from diverse populations was recorded, and the effect of each genomic variant was predicted with the Ensembl Variant Effect Predictor (VEP version 2.6)^22^ (appendix 1).

### Variant filtering

**Figure 2:**
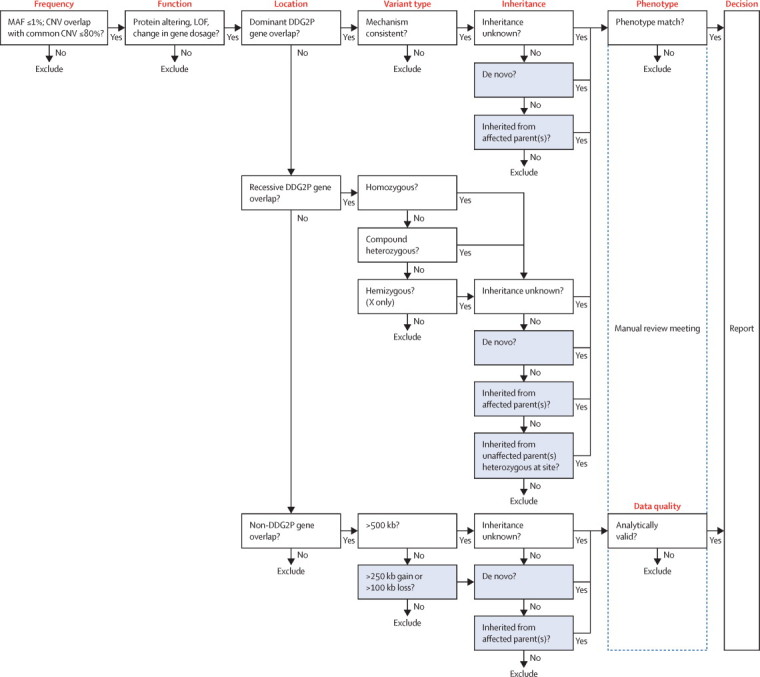
Variant filtering logic for clinical reporting within the study Genomic variants were filtered on the basis of six factors, of which the first five were automated and the final one was done manually: (1) frequency, prevalence of the variant in the general population (MAF ≤1%); (2) function, most severe predicted functional consequence, such as LOF, defined by specific sequence ontology terms (transcript ablation, splice donor variant, splice acceptor variant, stop-gained, frameshift variant, stop-lost, initiator codon variant, in-frame insertion, in-frame deletion, missense variant, transcript amplification, and coding sequence variant); (3) location, genomic location compared with DDG2P of published genes; (4) variant type, genotype (eg, heterozygous or homozygous) and loss or gain for small CNVs (which were only considered when they contained entire genes in which LOF or dominant negative mutations had been previously reported, and gains were only considered when they overlapped genes in which increased gene dosage mutations had been previously reported); (5) inheritance, aspects of the pipeline that are dependent on inheritance information derived from parental data are shaded; and (6) phenotype, patient phenotype was manually compared against published phenotypes for a particular gene. MAF=minor allele frequency. CNV=copy number variant. LOF=loss of function. DDG2P=Developmental Disorders Genotype-to-Phenotype database.

An automated variant filtering pipeline was used to narrow down the number of putative diagnostic variants (figure 2). First, common (>1% minor allele frequency) and non-functional (not protein-altering) variants were filtered out. Second, potentially pathogenic variants in known disease genes were selected in by comparison against an in-house database of genes consistently implicated in specific developmental disorders, the Developmental Disorders Genotype-to-Phenotype database (DDG2P). This database includes more than 1000 genes that have been consistently implicated in specific developmental disorders and is updated regularly with newly implicated genes (table 1; appendix 1; appendix 2). Each gene in DDG2P is associated with a specific developmental phenotype or syndrome via a particular genetic mechanism (autosomal dominant, autosomal recessive, or X-linked) and mutation consequence on the gene product (loss of function, activating mutation, increased gene dosage, etc). The use of DDG2P enabled any rare variant in a known DD gene with a predictable effect on the gene product to be flagged on the basis of inheritance, genotype, and likely mutational consequence. Large, rare CNVs overlapping non-DDG2P genes were also flagged based on a series of size thresholds (>100 kb for losses and >250 kb for gains where the inheritance was either de novo or segregated with disease, and >500 kb for any genic CNV for which the inheritance was unclear).

**Table 1:** Changes to DDG2P over time

	**July, 2012**	**November, 2012**	**July, 2013**	**November, 2013**
Total reportable genes*	819	875	1075	1128
Genes added	..	60	201	60
Genes removed†	..	4	1	7

In addition to genes being added or removed, annotations for existing genes can also change (eg, to include multiple modes or mechanisms). The November, 2013 version was used for the analysis presented here and includes 1128 reportable genes. DDG2P=Developmental Disorders Genotype-to-Phenotype database.

* DDG2P also contains non-reportable categories when there is insufficient evidence associating a gene and developmental disorder (appendix 1).

† The selection of variants for reporting is based on the strongest available evidence of gene function and no variants yet reported have been retracted because of changes in the DDG2P list.

### Sharing of results

Flagged variants were manually reviewed in a weekly multidisciplinary team meeting including clinical geneticists and genetic scientists to assess their analytical and clinical validity. Patients' detailed clinical presentation and family history were compared against published clinical features for each gene containing flagged variants, to evaluate the likely relevance in that specific patient. When there were sufficiently overlapping clinical features, the variant (chromosome, position, gene[s], allele, genotype, inheritance, and most severe predicted consequence) was approved for reporting to the regional genetics service via the patient's referring clinician. Variants were deposited into the patient's record via the secure study web portal DECIPHER, where they could be viewed in an interactive genome browser to enable local evaluation, diagnostic laboratory validation, and discussion with the family as appropriate. Anonymised variants were made publicly accessible after a short holding period (to ensure the opportunity for families to be informed before release). Full genomic datasets were also deposited in the European Genome-Phenome Archive in accordance with the REC approval for the study.

### Role of the funding source

The funders of the study did not contribute to the study design, data collection, data analysis, data interpretation, report writing, or the decision to submit this paper for publication. The corresponding author had full access to all the data in the study and final responsibility for the decision to submit for publication.

## Results

**Figure 3:**
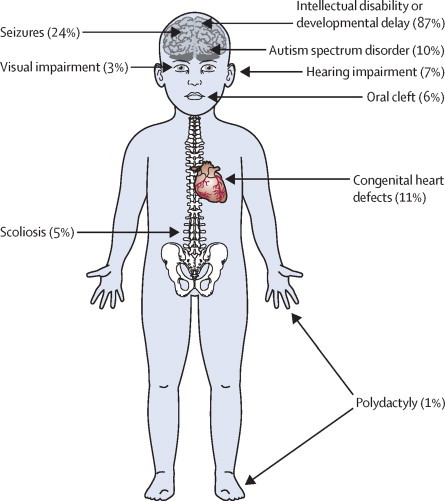
Representation of phenotypic diversity in cohort Our patient cohort represents children with a wide range of severe undiagnosed developmental disorders ascertained clinically across the UK.

To achieve equity of access for all undiagnosed families with developmental disorders in the British Isles, every UK NHS regional genetics service was involved in supporting and setting up the study; Ireland was subsequently added after the study had started. Almost all consultant clinical geneticists (more than 180) across all 24 regional genetics services in the UK and Ireland have recruited families to the DDD study, with the help of local research coordinators (typically research nurses or genetic counsellors). Around 2000 families were recruited in the first year of the study, rising to more than 8000 within 3 years. Among the first 1133 complete family trios (child, mother, and father), the male-to-female ratio among the probands was 51:49 and the median age at last clinical assessment was 5·5 years (SD 4·0, range 0–16). 121 (11%) children had one parent affected with a (typically milder) developmental phenotype, and 23 (2%) had both parents affected with developmental phenotypes (most often mild intellectual disability). Before entering DDD, 868 (77%) of the cohort had received clinical microarray testing, 633 (56%) had at least one targeted genetic test, and 522 (46%) had received both. Across the cohort, 1435 unique phenotype terms of the roughly 10 000 available in the HPO were used to describe clinical presentations, with a mode of 4 per proband (range 1–27); 987 (87%) children had intellectual disability, 270 (24%) had a history of seizures, and 121 (11%) had a congenital heart defect (figure 3).

**Figure 4:**
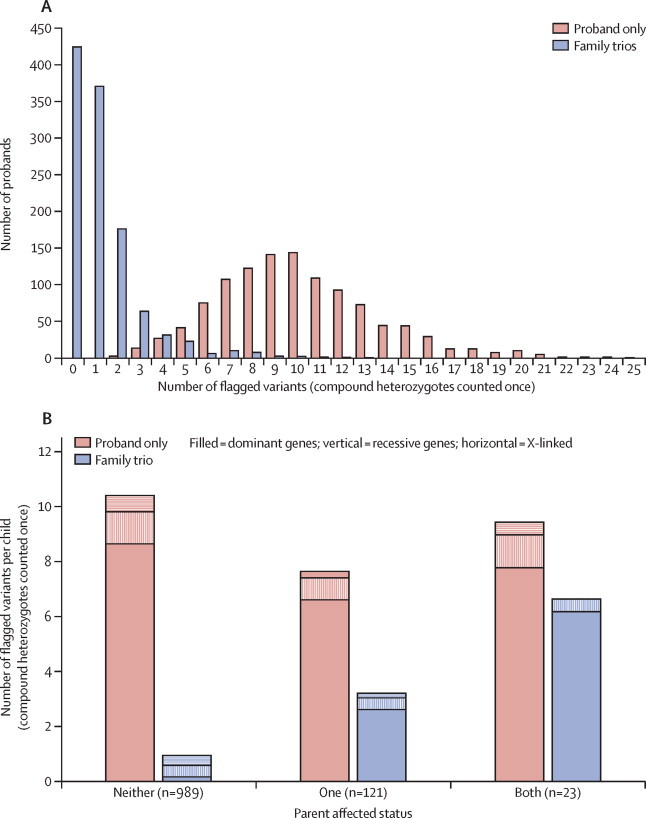
Analysis of flagged variants in all 1133 children excluding (red) and including (blue) filtering on the basis of parental genotypes and affected status (using the November 2013 version of DDG2P) (A) Histogram of the number of flagged single nucleotide variants and insertion-deletions in 1133 children with and without parental data. (B) Mean number of flagged variants per child with and without parental data for families where neither, one, or both parents are affected by a developmental phenotype, subdivided by DDG2P genetic mechanism. Note that compound heterozygous variants are counted once. Red=proband-only analysis. Blue=family-trio analysis with parental genotype data. Filled=autosomal dominant DDG2P genes. Vertical stripes=autosomal recessive DDG2P genes. Horizontal stripes=X-linked DDG2P genes. DDG2P=Developmental Disorders Genotype-to-Phenotype database.

Around 80 000 genomic variants were identified from exome sequencing and array comparative genomic hybridisation in each individual proband, of which on average 400 were rare and protein altering, and 30 of these overlapped known DDG2P genes. Further filtering based on DDG2P categories resulted in a median of 10 flagged SNVs and indels per proband in the absence of parental data (range 2–25). We further refined this to a median of 1 per proband (range 0–13) using inheritance information derived from parental data (figure 4). The difference between the number of variants with and without parental data is primarily due to heterozygous benign variants in dominant genes inherited from unaffected parents (table 2). Probands with unaffected parents had a mean of 0·9 (SD 0·9) variants flagged, which increased to 3·2 (2·4) with one affected parent and 7·4 (2·7) with two affected parents (figure 4). In addition to SNVs and indels, a further 0·2 CNVs per proband were flagged.

**Table 2:** Total number of single nucleotide variants and insertion-deletions flagged by the clinical reporting workflow in all 1133 probands (in the November, 2013 version of DDG2P) compared with the number of variants that would have been flagged in the same probands in the absence of parental data

	**Family-trio analysis**		**Proband-only analysis**
	Inherited	De novo*	
Autosomal dominant	473	193	9529
Autosomal recessive (homozygotes)	83	0	191
Autosomal recessive (compound heterozygotes)†	341	6	1071
X-linked dominant	38	21	269
X-linked recessive	322	15	387
Total	1257	235	11 447

Inherited variants in autosomal dominant DDG2P genes account for the main difference, in which only de novo variants and those inherited from an affected parent are likely to be of clinical interest. Around 90% of flagged variants were predicted to be missense point mutations. DDG2P=Developmental Disorders Genotype-to-Phenotype database.

* Before secondary validation by targeted Sanger sequencing.

† Two or more likely pathogenic variants in different copies of the same gene, counted once per compound variant.

All flagged variants were automatically annotated with pathogenicity scores from two variant prioritisation algorithms (SIFT^23^ and PolyPhen^24^) and compared against the public Human Gene Mutation Database (HGMD) and the Leiden Open Variation Database (LOVD), which together contained only 14% of flagged variants. The phenotypes recorded for each patient were compared against those previously published for patients with similar mutations in the same gene, using primarily DDG2P, PubMed, GeneReviews, and OMIM. If the patient's phenotype was deemed to be inconsistent with the genetic change (on the basis of current knowledge of the phenotypic spectrum of that gene), the variant was not returned to the patient's clinician. The manual review is the most human-intensive step of the workflow, but was essential for validation and improvement of the automated filtering and prevention of likely non-pathogenic and incidental findings from being reported. We expedited the review of the variants in the most frequently observed genes by requiring specific clinical features (eg, deafness) that related to these genes to be noted in the patient. Recent work has highlighted the need for caution in interpretation of X-linked causes of intellectual disability,^25^ and we made the decision not to consider any inherited missense variants on the X-chromosome (except in patients with a family history of developmental disorders or when the variant itself was present in either HGMD or LOVD) because of the large number of variants but low prior probability of causality for this class of variation.

After automated variant filtering, we manually reviewed 1696 candidate variants in 1133 family trios and reported 317 likely diagnostic SNVs, indels, and CNVs (table 3). Many of these diagnostic variants have subsequently been validated in an accredited diagnostic laboratory and communicated to individual families. Additionally, we found six likely pathogenic cases of uniparental disomy^26^ and five large mosaic chromosomal rearrangements (unpublished data). We reported two separate genetic findings in 17 individual cases in which both findings might contribute to the phenotype.

**Table 3:** Likely diagnoses in the first 1133 families

		**Reviewed (SNV, CNV)**	**Reported as likely diagnostic**	**Predictive value of flag (%)**	**Diagnostic yield (%; n=1133)**
Autosomal dominant					
	De novo	242 (193, 49)	184	75%	16%
	Inherited	528 (473, 55)	27	5%	2%
Autosomal recessive					
	De novo	6 (6, 0)	0	..	..
	Inherited	425 (424, 1)	52	13%	5%
X-linked					
	De novo	41 (36, 5)	31	75%	3%
	Inherited	371 (360, 11)	23	6%	2%
Uncertain inheritance		83 (0, 83)	0	..	..
Chromosomal events					
	Uniparental disomy	..	6	..	0·5%
	Mosaicism	..	5	..	0·5%
Total		1696	328	19%	27%*

See appendix 2 for details of individual genes and phenotype classes. The predictive value is the probability that a flagged variant was reported as likely diagnostic (reported or reviewed), and the diagnostic yield is the contribution of that type of variant to the overall diagnostic yield. Note that three pairs of siblings and two pairs of monozygotic twins received the same diagnosis. Only 14% of reported variants were present in the public Human Gene Mutation Database or Leiden Open Variation Database; 84% of flagged variants present in these databases were not reported, because they did not appear to be relevant to the child's phenotype. SNV=single nucleotide variant. CNV=copy number variant.

* 17 probands received two contributory pathogenic variants.

With 328 likely diagnostic or strongly contributory variants in 311 of 1133 children, our overall diagnostic rate is 27%. Within DDG2P genes, de novo variants accounted for 65% of our diagnoses across the cohort, and 92% of those that were validated were regarded as pathogenic (table 3). Although some genes were hit multiple times, we found a diagnosis in only 146 (13%) individual DDG2P genes, of which 92 were hit only once in this cohort (appendix 2). We assessed the performance of five variant prioritisation tools^23^, ^24^, ^27^, ^28^, ^29^ to help to interpret the pathogenicity of missense variants (90% of flagged variants), and found that PolyPhen and MutationTaster discriminated equally well between reported and non-reportable variants (appendix 1), but were nonetheless unable to predict the likely diagnostic variants accurately.

## Discussion

**Figure 5:**
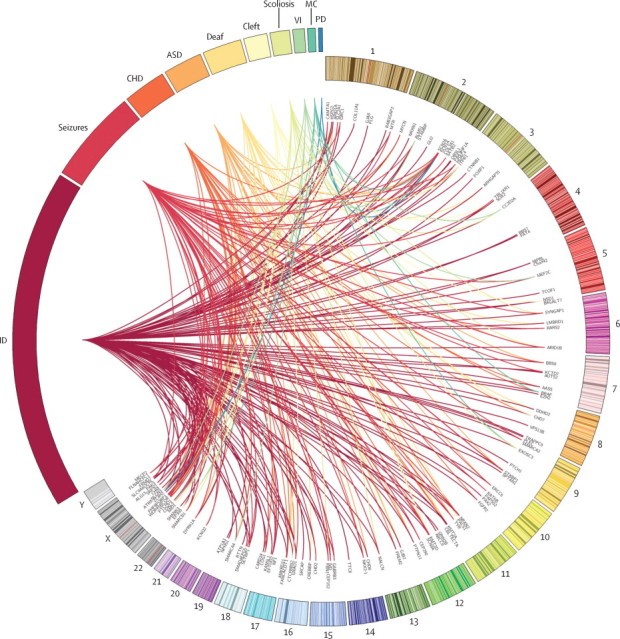
Genetic diagnoses associated with broad phenotype categories Circos-style plot representing the genetic heterogeneity within developmental disorders, showing individual diagnoses in known Developmental Disorders Genotype-to-Phenotype database genes, which links the genomic location of each gene with some key phenotypes in each child. Phenotypes are listed outside the widest arc of the circle, chromosome numbers are indicated outside the smaller arc, and individual gene names are listed inside. Links are coloured by phenotype group. See appendix 2 for details of the diagnoses. ID=intellectual disability. CHD=congenital heart defect. ASD=autism spectrum disorders. Deaf=hearing impairment. Cleft=oral cleft. VI=visual impairment. MC=microcephalic dwarfism. PD=polydactyly.

We have developed and implemented a scalable workflow within a large-scale rare-disease research study to allow return of clinically pertinent genetic variants to clinicians and research participants (panel). The workflow is consistent with recently recommended guidelines for investigating causality of sequence variants in human disease,^34^, ^35^ and we hope that it will act as a prototype for the translation of diagnostic genome sequencing into the clinic for a range of rare diseases. The semi-automated system we have described achieved a diagnostic yield of 27% in previously investigated, yet undiagnosed, children with developmental disorders caused by variants in known genes across the genome (figure 5; appendix 1). The system is amenable to an iterative approach to reanalysis of patient data, and we expect that our diagnostic yield will increase in the coming years as a result of novel gene discovery (both within^36^ and outside of the DDD study) and ongoing improvements to the analysis algorithms and variant filtering rules.

Panel **Research in context**

**Systematic review**

Trio exome sequencing is a highly successful research tool for new gene discovery.^2^ A recent health technology assessment concluded that there is “scarce evidence supporting the use of whole exome sequencing for etiologic diagnosis in patients with ID/DD”, and warned that this technology presents “ethical dilemmas related to incidental findings in the analysis of genetic material in these patients”.^30^ A systematic review was not done, but we are aware of two smaller studies that have shown the likely usefulness of trio exome sequencing for clinical diagnosis of children with severe intellectual disability, including 51 patients from Germany or Switzerland^31^ and 100 patients from Nijmegen (Netherlands).^5^ Clinical investigation of 410 rare disease patients from the University of California^32^ achieved a 31% diagnostic rate for trio-based exome sequencing, and a large single-centre study of 2000 rare disease patients from Baylor (USA)^33^ reported a diagnostic rate of 25% using exome sequencing of the proband followed by targeted follow-up testing in the parents.

**Interpretation**

Deciphering Developmental Disorders (DDD) is the first nationwide exome sequencing study. It involves more than 1000 children with undiagnosed developmental disorders and their parents, combining genome-wide data from high-resolution microarrays and trio exome sequencing to maximise detection of potentially pathogenic variants. It achieved a consistent diagnostic rate while demonstrating a scalable, collaborative model for translational research. The informatics workflow developed by DDD has addressed one of the key ethical challenges raised by massively parallel sequencing technologies and, by using a clinically targeted analysis, shown that incidental findings can be minimised. The DDD study will continue to recruit throughout the UK and Ireland until April, 2015, and aims to reach 12 000 patients; we expect to continue to improve our analysis workflow and increase the diagnostic rate higher than 30% as novel causal genes are discovered and incorporated into diagnostic analyses, allowing existing patient data to be reinterpreted.

De novo variants had by far the highest predictive value and diagnostic yield in our cohort, highlighting the value of having genotype data from both parents as well as the child. Of our 215 likely de novo diagnoses, 41 were CNVs and 174 were SNVs or indels, of which around half (90/174) were novel missense variants and half (84/174) were likely loss-of-function variants. Although we attempted to validate almost all putative de novo SNVs and indels in our cohort using targeted Sanger sequencing—the diagnostic gold standard—this process sometimes needed several attempts to optimise primer design and achieve high-quality data. Given appropriate quality thresholds and read depths in all three family members, we believe that trio exome sequencing is highly accurate for assessing de novo mutations, but that targeted Sanger validation will remain important for diagnostic confirmation and cascade testing.

For families in which neither parent is affected by the same disorder, sequencing of parent–child trios rather than individual probands offers around a ten-times reduction in the number of candidate variants, thus substantially increasing the speed and likelihood of reaching an accurate diagnosis. By contrast, when one or both parents are similarly affected, trio sequencing offers only a three-times or 1·5-times reduction, respectively, and might therefore be less informative. By contrast with our trio-sequencing approach, de novo variants could instead be identified in a stepwise fashion by exome sequencing of the proband, then assay of possibly pathogenic variants in both parents by targeted Sanger sequencing. However, in view of the rapidly decreasing cost of exome and genome sequencing (currently £1000–5000 per individual genome), trio exome sequencing could offer a more rapid, cost-effective, and scalable diagnostic method in addition to providing increased usefulness in research.

Since most of our cohort had received at least one genetic test before entering the study, introduction of exome sequencing early in the diagnostic pathway could substantially reduce costs and increase diagnostic yields versus current clinical practice. Additionally, a small but growing number of childhood developmental disorders are amenable to existing therapeutic interventions, and early treatment offers substantial benefits in preventing irreversible clinical manifestations of the condition. Currently, 82 reportable DDG2P genes are associated with inborn errors of metabolism, which are causally related to intellectual disability, and are potentially amenable to therapy.^37^ To date, five DDD children have a diagnostic variant in one of these treatable ID genes (*DHCR7, IVD, LMBRD1, MTR,* and *SLC2A1*) and might be suitable for either dietary restriction, supplementation, or pharmacological intervention.

When developing our clinical feedback policy, our aim was to maximise likely diagnoses while minimising incidental findings, and we were conscious of the fact that clinical teams have neither the resources nor the remit to attempt to validate multiple variants of uncertain significance in every patient. Like any medical test, the variant filtering process necessitates a trade-off between sensitivity and specificity, and any change to the analytical pipeline will potentially alter this balance. With an ever-expanding set of genes implicated in developmental disorders, and in light of the fact that around 80% of our flagged variants did not appear to be relevant to the child's developmental disorder, we hope to use the manually curated results presented here to refine the variant filtering rules further. Specifically, we expect to be able to lower the frequency threshold to 0·1% for dominantly inherited variants and, following an analysis of variant prioritisation methods (appendix 1), to use PolyPhen to exclude low-scoring inherited missense variants predicted to be benign. Although we plan to automate more of the clinical reporting process, we expect that some variants will always need expert gene-specific clinical and scientific interpretation.

Because of the large number of rare variants in every genome that are unrelated to disease, a genotype–phenotype database (such as DDG2P) is crucial to allow novel variants to be prioritised on the basis of current knowledge of gene–disease associations and allelic requirements. In principle this approach could be applied to any medical specialism for the diagnosis of highly penetrant genetic conditions. Although it is possible to use a generic genotype-to-phenotype database for variant filtering based on a broad phenotype (ie, developmental disorders), detailed phenotypes are crucial for assignment of likely pathogenicity to candidate variants. Fewer candidate variants could be flagged by use of a smaller, more targeted list, and restriction of the assay to only this list of genes could potentially reduce the cost. We used the DDG2P database to allow return of variants when the patient's phenotypes, developmental milestones, and morphometric data were consistent with published reports (this approach was not intended to allow association of new phenotypes with known genes, which will need further research). However, the value of whole genome or exome sequencing as compared with targeted gene panels lies primarily in the research potential for gene discovery,^36^ and the clinical usefulness of enabling future diagnoses to be made in current patients from existing data. This diagnostic benefit is already apparent within our cohort, in which, on average, 20 new disease-implicated genes are published and added to DDG2P per month, making iterative analysis across the entire genomic dataset, coupled with automated variant filtering and re-reviewing, essential to maximise the diagnostic benefit.

An outstanding question remains regarding whether researchers should actively search for so-called incidental findings^38^ and thus undertake genomic screening of medically relevant genomic variation as recommended in standard clinical practice by the American College of Medical Genetics and Genomics.^39^ We note that searching for incidental findings in this manner is a choice not a prerequisite of whole-genome sequencing, because the analysis can be entirely diagnostically targeted (as we have shown). At the outset of DDD, we could not be confident that actively screening for additional incidental findings unrelated to the child's developmental disorder would be in the best interests of the patients and families within DDD, particularly in view of the paucity of data regarding the true significance of such variants ascertained in individuals with no known previous risk of the cognate disease. Additionally, such screening could potentially undermine established clinical practice with respect to screening of children for adult-onset conditions.

Nonetheless, furnished with supplementary genotype-to-phenotype databases or specific variant lists, the analytical procedure we have described could be adapted to allow simultaneous opportunistic screening for many conditions, if this were deemed to be ethically appropriate and evaluation showed that there was evidence of benefit to support this approach.^8^ However, the infrastructure needed to create and support this workflow is considerable, and substantial investment of staff time at all levels is needed to ensure that it is accurate and robust. Hundreds of individuals were involved in data entry, generation, management, processing, analysis, interpretation, and dissemination within DDD, including around 15 full-time staff with a dedicated laboratory and priority access to a high-performance computing cluster. Around 75 h were spent manually reviewing the flagged variants in meetings that were attended by between two and eight people including at least one consultant clinical geneticist, and assessing the clinical relevance of rare functional variants even in well-known developmental disorder genes was often very challenging. Interpretation of variants relating to many other diseases in the absence of known symptoms or family history would be even harder, particularly in the absence of robust data concerning population-ascertained penetrance, and additional disease-specific expertise would no doubt be needed. For these reasons, we chose to focus on identifying pertinent findings within the DDD study to maximise diagnostic yield and drive research into the underlying causes of developmental disorders.
