# Supplementary material for: Genetic diagnosis of developmental disorders in the DDD study: a scalable analysis of genome-wide research data
Source: Lancet. 2015 Apr 4;385(9975):1305–14. doi: 10.1016/S0140-6736(14)61705-0 (PMC4392068; doi:10.1016/S0140-6736(14)61705-0)
Supplement: Supplementary appendix 1 [file mmc1.pdf]

THE LANCET

Supplementary appendix

This appendix formed part of the original submission and has been peer reviewed. We post it as supplied by the authors.

Supplement to: Wright CF, Fitzgerald TW, Jones WD, et al, on behalf of the DDD study. Genetic diagnosis of developmental disorders in the DDD study: a scalable analysis of genome-wide research data. *Lancet* 2015; published online Dec 17. http://dx.doi.org/10.1016/S0140-6736(14)61705-0.

APPENDIX 1

Supplementary Materials

S1. Detailed Methods

Sample Collection
Saliva samples were taken from patients and their parents using barcoded Oragene®DNA collection kits (DNA Genotek), and sent to the Wellcome Trust Sanger Institute (WTSI), where genomic DNA was extracted using a QIAsymphony instrument; blood-extracted genomic DNA from the proband was also provided by regional molecular genetics laboratories. A bespoke Laboratory Information Management System (LIMS) was developed, which interacted with DECIPHER to facilitate sample tracking. Following gel electrophoresis, an automated volume check (BioMicroLab) and assessment of concentration via a pico green assay (Beckman FX, NX-96, Molecular devices DTX reader). Fifty-eight unique single nucleotide polymorphisms (SNPs) were genotyped by the WTSI core sample logistics facility using Sequenom® mass spectrometry to provide a 'molecular barcode' for sample/data tracking; any inconsistent family relationships were identified and resolved prior to genomic analysis either via tube swaps or individual sample failures.

Genomic Analysis
Copy number variation was initially assessed in the probands by the DDD microarray team using a custom Agilent 2x1M CGH array (Amadid No.s 031220/031221), with 5 probes per exon and a mean backbone spacing of ~4kb. Patient samples were processed in batches of 95 using an Agilent BRAVO robot, labelled with Cy5 with Agilent SureTag labelling reagents, and hybridised against a pool of 500 developmentally normal males labelled with Cy3. All laboratory procedures were supported by our bespoke LIMS. Arrays were scanned in an Agilent G2565CA scanner at a 3μm resolution, and processed using Feature Extraction v10.5.1.1. CNVs were then called using a novel in-house analysis package (CNsolidate, manuscript in preparation) that combines 12 weighted change point detection algorithms. Patient data were annotated with allele frequencies based on overlap with a set of controls analysed on the same platform (570 population controls from the UK Blood Service and 455 developmental normal individuals from the Scottish Family Health Study). The WTSI genotyping team also genotyped the first ~1000 families using the Illumina 700K OmniExpress SNP-array (SangerDDD_OmniExPlusv1_15019773_A) with an additional 100K custom content to fill any large gaps. The inheritance of rare CNVs identified in the proband using aCGH was subsequently assessed on the SNP-array using a Bayesian framework.

Exome sequencing of family trios was performed by the WTSI core pull-down and sequencing teams, using a custom Agilent SureSelect Exome bait design (Agilent Human All Exon V3 Plus with custom ELID # C0338371), 8-plex sample multiplexing and an Illumina HiSeq with 4 samples per lane and a mean depth of 50X. The exome targeted 58.62 Mb (271,064 baits) of which 51.64 Mb (213,384 baits) consisted of exonic targets (39 Mb) and their flanking regions, 6.9 Mb (57680

baits) of regulatory regions were targeted using custom baits. The median (n=3,399) average sequencing depth (ASD = bases sequenced/bases targeted) was 90X across the whole targeted sequence or 93X across autosomal targets only. 95% of all samples had an average sequencing depth higher than 63X. At least 90% of all targeted regions have a median ASD higher than 15X. Only 16026 baits showed a median ASD smaller than 10X, comprising 800kb of protein coding sequence. More than 85% of all probes were consistently covered (ASD >10X) across the three samples of the trio in at least 90% of the 1133 trios. Alignment was performed using BWA[1] and variants were called from BAM files using GATK[2], SAMtools[3] and Dindel[4]. Putative *de novo* variants were identified from trio BAM files using DeNovoGear[5] and CNVs were called using a novel in-house algorithm (CoNVex, manuscript in preparation). Variant Call Format (VCF) files from each variant calling pipeline were then merged and annotated with the most severe consequence predicted by Ensembl Variant Effect Predictor (VEP version 2.6)[6], and minor allele frequencies from a combination of the 1000 Genomes project (www.1000genomes.org), UK10K (www.uk10k.org), the NHLBI Exome Sequencing Project (esp.gs.washington.edu), Scottish Family Health Study (www.generationscotland.org), UK Blood Service and unaffected DDD parents. Putative *de novo* SNVs and indels were validated in-house using whole genome amplified DNA, PCR and capillary sequencing.

Data Filtering and Sharing
Rare (minor allele frequency $\leq$0.01%) and protein-altering coding variants (defined by the sequence ontology terms: transcript ablation, splice donor variant, splice acceptor variant, stop-gained, frameshift variant, stop-lost, initiator codon variant, in-frame insertion, in-frame deletion, missense variant, transcript amplification and coding sequence variant) in individual probands were compared against an in-house developmental disorder genotype-to-phenotype database (DDG2P) and the patient's clinical features. Flagged variants were manually reviewed and likely diagnostic variants fed-back to the referring clinical geneticist via the patient's record in DECIPHER, where they can be viewed in an interactive genome browser by all members of the regional genetics service to enable local clinical evaluation, diagnostic laboratory validation and discussion with the family as appropriate. These variants are made publicly accessible after a short holding period (to ensure families are informed). Full genomic datasets are also deposited in EGA (www.ebi.ac.uk/ega) for future research.

References

1. Li H, Durbin R. Fast and accurate short read alignment with Burrows-Wheeler transform. Bioinformatics. 2009; **25**(14): 1754-60.
2. McKenna A, Hanna M, Banks E, *et al*. The Genome Analysis Toolkit: A MapReduce framework for analyzing next-generation DNA sequencing data. Genome Research. 2010; **20**(9): 1297-303.
3. Li H, Handsaker B, Wysoker A, *et al*. The Sequence Alignment/Map format and SAMtools. Bioinformatics. 2009; **25**(16): 2078-9.
4. Albers CA, Lunter G, MacArthur DG, *et al*. Dindel: Accurate indel calls from short-read data. Genome Research. 2011; **21**: 961-73.
5. Ramu A, Noordam MJ, Schwartz RS, *et al*. DeNovoGear: de novo indel and point mutation discovery and phasing. Nat Meth. 2013; **10**(10): 985-7.

6. McLaren W, Pritchard B, Rios D, *et al*. Deriving the consequences of genomic variants with the Ensembl API and SNP Effect Predictor. Bioinformatics. 2010; **26**(16): 2069-70.
7. Robin X, Turck N, Hainard A, *et al*. pROC: an open-source package for R and S+ to analyze and compare ROC curves. BMC Bioinformatics. 2011; **12**(1): 77.

S2. DDG2P

Overview
The DDG2P dataset integrates data on genes, variants and phenotypes relating to developmental disorders. It is constructed entirely from published literature, and is primarily an inclusion list to allow targeted filtering of genome-wide data for diagnostic purposes in the DDD study. The database was compiled with respect to published genes, and annotated with types of disease-causing gene variants. Each row of the database associates a gene with a disease phenotype via an evidence level, inheritance mechanism and mutation consequence. Some genes therefore appear in the database more than once, where different genetic mechanisms result in different phenotypes. DDG2P is produced and curated by UK consultant clinical geneticists. It is regularly updated and the dataset used here is from 7th November 2013 (available in Appendix 2).

Establishing and updating DDG2P
DDG2P is dependent on the RefSeq list of human genes downloaded from USCS Genome Informatics Table Browser interface (genome.ucsc.edu). The initial assignment of developmental disease genes, allele requirements and mutational consequence within DDG2P was achieved using three main sources: a systematic review of all articles in the previous 5 years of Nature Genetics and American Journal of Human Genetics by HVF and DRF; a review of all diagnostic tests currently offered by NHS DNA diagnostic laboratories in the UK; and text searching of the "Involvement in Disease" topic within the "General Annotation" field of UniProt (www.uniprot.org) using multiple different Boolean combinations of terms related to developmental disorders. The database interfaces with OMIM to inform the curation process. DDG2P is currently maintained as a Filemaker Pro relational database and is updated via regular review of the literature and from presentations at scientific meetings attended by DRF and HVF. All new gene-disease entities are added to the database by DRF and are made public via Twitter using @DDG2P, and regular releases are available via DECIPHER (https://decipher.sanger.ac.uk).

Definitions of terms and evidence base used in DDG2P
The definitions of the categorical terms used for assigning the DDG2P status of a gene, the allele requirements of each disease linked to a gene and the mutational consequence associated with each allele requirement are given in Appendix 2, Tables S1a, S1b and S1c respectively. The main evidence source used in DDG2P is the PMID of peer-reviewed articles that have been reviewed in order to categorise each gene-disease (one-to-many) relationship. The November 2013 version of DDG2P is provided in Table S1d of Appendix 2.

S3. DDD Consortium Members

Central DDD Team
Wellcome Trust Sanger Institute, Wellcome Trust Genome Campus, Hinxton, Cambridge, CB10 1SA, UK
&
The Ethox Centre, Nuffield Department of Population Health, University of Oxford, Old Road Campus, Oxford, OX3 7LF, UK

*DDD Management Team (*Principal Investigator)*
Jeffrey C. Barrett, Nigel P. Carter, Helen V. Firth, David R. FitzPatrick , Matthew E. Hurles*, Michael Parker, Caroline F. Wright

DDD Laboratory Team
Kirsty Ambridge, Daniel M. Barrett, Tanya Bayzetinova, Susan Gribble, Netravathi Krishnappa, Laura E. Mason, Elena Prigmore, Diana Rajan

DDD Model Organisms
Eve L. Coomber, Sebastian S. Gerety

DDD Informatics Team
Stephen Clayton, Tomas W. Fitzgerald, Philip Jones, Ray Miller, Adrian R. Tivey

DDD Analysis Team
Nadia Akawi, Saeed Al-Turki, Jeffrey C. Barrett, Tomas W. Fitzgerald, Matthew E. Hurles, Wendy D. Jones, Daniel King, Margriet van Kogelenberg, Jeremy McRae, Katherine I. Morley, Vijaya Parthiban, Alejandro Sifrim

DDD Ethics, Social Science and Policy Team
Anna Middleton, Michael Parker, Caroline F. Wright

Central Wellcome Trust Sanger Institute Staff
Wellcome Trust Sanger Institute, Wellcome Trust Genome Campus, Hinxton, Cambridge, CB10 1SA, UK

DECIPHER Team
A. Paul Bevan, Eugene Bragin, G. Jawahar Swaminathan

WTSI Pipelines Staff (sample QC, genotyping, pulldown, sequencing, informatics)
Rob Andrews, John Burton, Suzannah J. Bumpstead, Sarah Edkins, Peter Ellis, Emma Gray, David Jones, Carol Scott, Douglas Simpkin, Danielle Walker, Sara Widaa

WTSI FISH Team
Ruby Banerjee, Beiyuan Fu, Sandra Louzada Gomes Pereira, Fentang Yang,

GENERATION SCOTLAND
University of Edinburgh, Institute of Genetics & Molecular Medicine, Western General Hospital, Crewe Road South, Edinburgh, EH4 2XU, UK

Scottish Family Health Study
Anna Dominiczak, Andrew Morris, David Porteous, Blair Smith

UK NHS Regional Genetic Services ***(* local Principal Investigator)***

Aberdeen (North of Scotland Regional Genetics Service, NHS Grampian, Department of Medical Genetics Medical School, Foresterhill, Aberdeen, AB25 2ZD, UK)
Recruiting Consultant Clinical Geneticists: John Dean*, Ruth McGowan, Alison Ross
Research Nurse/ Genetic Counsellors: Mariella D'Alessandro
Diagnostic Laboratory scientists: Paul Batstone, Shalaka Samant

Belfast (Northern Ireland Regional Genetics Centre, Belfast Health and Social Care Trust, Belfast City Hospital, Lisburn Road, Belfast, BT9 7AB, UK)
Recruiting Consultant Clinical Geneticists: Tabib Dabir, Deirdre Donnelly, Alex Magee, Vivienne McConnell, Shane McKee*, Fiona Stewart,
Research Nurse/ Genetic Counsellors: Claire Kirk
Diagnostic Laboratory scientists: Mervyn Humphreys, Susan McNerlan

Birmingham (West Midlands Regional Genetics Service, Birmingham Women's NHS Foundation Trust, Birmingham Women's Hospital, Edgbaston, Birmingham, B15 2TG, UK)
Recruiting Consultant Clinical Geneticists: Louise Brueton, Trevor Cole*, Nicola Cooper, Helen Cox, Joanna Jarvis, Derek Lim , Jenny Morton, Andrew Norman, Chirag Patel, Nicola Ragge, Saba Sharif, Mark Tein , Julie Vogt , Denise Williams
Research Nurse/ Genetic Counsellors: Gail Kirby
Diagnostic Laboratory scientists: David Bohanna, Kirsten McKay, Dominic J McMullan

Bristol (Bristol Genetics Service (Avon, Somerset, Gloucs and West Wilts), University Hospitals Bristol NHS Foundation Trust, St Michael's Hospital, St Michael's Hill, Bristol, BS2 8DT, UK)
Recruiting Consultant Clinical Geneticists: Ruth Newbury-Ecob*, Sarah Smithson
Research Nurse/ Genetic Counsellors: Rose Hawkins
Diagnostic Laboratory scientists: Eileen Roberts, Christopher Wragg

Cambridge (East Anglian Medical Genetics Service, Box 134, Cambridge University Hospitals NHS Foundation Trust, Cambridge Biomedical Campus, Cambridge ,CB2 0QQ, UK)
Recruiting Consultant Clinical Geneticists: Ruth Armstrong, Helen Firth*, Simon Holden, Sarju Mehta, Soo-Mi Park, Joan Paterson, Lucy Raymond, Richard Sandford, Geoff Woods
Research Nurse/ Genetic Counsellors: Jonathan Roberts, Sarah Wilcox
Diagnostic Laboratory scientists: Ingrid Simonic, Becky Treacy

Cardiff (Institute Of Medical Genetics, University Hospital Of Wales, Heath Park, Cardiff, CF14 4XW, UK and Department of Clinical Genetics, Block 12, Glan Clwyd Hospital, Rhyl, Denbighshire, LL18 5UJ, UK)

Recruiting Consultant Clinical Geneticists: Hayley Archer, Sally Davies, Dhavendra Kumar, Emma McCann*, Daniela T. Pilz*, Annie Procter
Research Nurse/ Genetic Counsellors: Karenza Evans
Diagnostic Laboratory scientists: Sian Morgan, Hood Mugalaasi

Dublin (National Centre for Medical Genetics, Our Lady's Children's Hospital, Crumlin, Dublin 12, Ireland)
Recruiting Consultant Clinical Geneticists: Sally Ann Lynch*
Research Nurse/ Genetic Counsellors: Rosie O'Shea

Dundee (East of Scotland Regional Genetics Service, Human Genetics Unit, Pathology Department, NHS Tayside, Ninewells Hospital, Dundee, DD1 9SY, UK)
Recruiting Consultant Clinical Geneticists: Jonathan Berg*, David Goudie, Susann Schweiger
Research Nurse/ Genetic Counsellors: Debbie Rice
Diagnostic Laboratory scientists: David Baty, Norman Pratt

Edinburgh (MRC Human Genetics Unit, MRC IGMM, University of Edinburgh, Western General Hospital, Edinburgh, EH4 2XU, UK)
Recruiting Consultant Clinical Geneticists: David R. FitzPatrick*, Wayne Lam, Anne Lampe
Research Nurse/ Genetic Counsellors: Philip Greene
Diagnostic Laboratory scientists: Eddy Maher, David Moore

Exeter (Peninsula Clinical Genetics Service, Royal Devon and Exeter NHS Foundation Trust, Clinical Genetics Department, Royal Devon & Exeter Hospital (Heavitree), Gladstone Road, Exeter, EX1 2ED, UK)
Recruiting Consultant Clinical Geneticists: Carole Brewer, Bruce Castle, Emma Kivuva*, Julia Rankin, Charles Shaw-Smith, Claire Turner, Peter Turnpenny
Research Nurse/ Genetic Counsellors: Gemma Devlin, Sarah Everest
Diagnostic Laboratory scientists: Sian Ellard, Carolyn Tysoe

Glasgow (West of Scotland Regional Genetics Service, NHS Greater Glasgow and Clyde, Institute Of Medical Genetics, Yorkhill Hospital, Glasgow, G3 8SJ, UK)
Recruiting Consultant Clinical Geneticists: Rosemarie Davidson, Carol Gardiner, Shelagh Joss, Esther Kinning, Victoria Murday, John Tolmie*, Margo Whiteford
Research Nurse/ Genetic Counsellors: Alexis Duncan
Diagnostic Laboratory scientists: Gordon Lowther, Nicola Williams

Leeds (Yorkshire Regional Genetics Service, Leeds Teaching Hospitals NHS Trust, Department of Clinical Genetics, Chapel Allerton Hospital, Chapeltown Road, Leeds, LS7 4SA, UK)
Recruiting Consultant Clinical Geneticists: Chris Bennett, Moira Blyth*, Emma Hobson, Alison Kraus, Katrina Prescott*, Audrey Smith, Jenny Thomson
Research Nurse/ Genetic Counsellors: Miranda Squires
Diagnostic Laboratory scientists: Andrea Coates, Sarah Hewitt, Paul Roberts

Leicester (Leicestershire Genetics Centre, University Hospitals of Leicester NHS Trust, Leicester Royal Infirmary (NHS Trust), Leicester, LE1 5WW, UK)
Recruiting Consultant Clinical Geneticists: Pradeep Vasudevan*

Research Nurse/ Genetic Counsellors: Beckie Kaemba, Sandra Kazembe
Diagnostic Laboratory scientists: Lara Cresswell

Liverpool (Merseyside and Cheshire Genetics Service, Liverpool Women's NHS Foundation Trust, Department of Clinical Genetics, Royal Liverpool Children's Hospital Alder Hey, Eaton Road, Liverpool, L12 2AP, UK)
Recruiting Consultant Clinical Geneticists: Astrid Weber*, Alan Fryer, Lynn Greenhalgh, Elizabeth Sweeney
Research Nurse/ Genetic Counsellors: Gillian Roberts, Vivienne Sutton
Diagnostic Laboratory scientists: Angela Douglas, Una Maye

London - North West Thames (North West Thames Regional Genetics Centre, North West London Hospitals NHS Trust, The Kennedy Galton Centre, Northwick Park And St Mark's NHS Trust Watford Road, Harrow, HA1 3UJ, UK)
Recruiting Consultant Clinical Geneticists: Birgitta Bernhard, Angela Brady, Natalie Canham*, Neeti Ghali, Susan Holder, Anthony Vandersteen Emma Wakeling
Research Nurse/ Genetic Counsellors: Cheryl Sequeira, Roldan Singzon
Diagnostic Laboratory scientists: Louise Bourdon, Stewart Payne

London - Great Ormond Street (North East Thames Regional Genetics Service, Great Ormond Street Hospital for Children NHS Foundation Trust, Great Ormond Street Hospital, Great Ormond Street, London, WC1N 3JH, UK)
Recruiting Consultant Clinical Geneticists: Jane Hurst*, Melissa Lees , Elisabeth Rosser, Richard Scott
Research Nurse/ Genetic Counsellors: Kate Brunstrom, Georgina Hollingsworth
Diagnostic Laboratory scientists: Lucy Jenkins, Jonathon Waters

London - Guy's (South East Thames Regional Genetics Centre, Guy's and St Thomas' NHS Foundation Trust, Guy's Hospital, Great Maze Pond, London, SE1 9RT, UK)
Recruiting Consultant Clinical Geneticists: Fiona Connell, Charu Deshpande, Frances Flinter, Melita Irving, Dragana Josifova, Shehla Mohammed*, Leema Robert
Research Nurse/ Genetic Counsellors: Tina Fendick, Caroline Langman
Diagnostic Laboratory scientists: Caroline Ogilvie, Michael Yau

London - St George's (South West Thames Regional Genetics Centre, St George's Healthcare NHS Trust, St George's, University of London, Cranmer Terrace, London, SW17 0RE, UK)
Recruiting Consultant Clinical Geneticists: Frances Elmslie, Tessa Homfray, Sahar Mansour*, Meriel McEntagart, Anand Saggar, Kate Tatton-Brown
Research Nurse/ Genetic Counsellors: Uruj Anjum
Diagnostic Laboratory scientists: Karen Marks, Rohan Taylor

Manchester (Manchester Centre for Genomic Medicine, St Mary’s Hospital, Central Manchester University Hospitals NHS Foundation Trust, Manchester Academic Health Science Centre, Manchester M13 9WL)
Recruiting Consultant Clinical Geneticists: Kate Chandler, Jill Clayton-Smith*, Yanick Crow, Elizabeth Jones, Bronwyn Kerr, Kay Metcalfe
Research Nurse/ Genetic Counsellors: Carina Donnelly, Zara Skitt
Diagnostic Laboratory scientists: Lorraine Gaunt, Emma Miles

Newcastle (Northern Genetics Service, Newcastle upon Tyne Hospitals NHS Foundation Trust, Institute of Human Genetics, International Centre for Life, Central Parkway, Newcastle upon Tyne, NE1 3BZ, UK)
Recruiting Consultant Clinical Geneticists: John Burn, Richard Fisher, Judith Goodship , Alex Henderson, Tara Montgomery, Miranda Splitt*, Michael Wright
Research Nurse/ Genetic Counsellors: Linda Sneddon
Diagnostic Laboratory scientists: David Bourn, Stephen Hellens

Nottingham (Nottingham Regional Genetics Service, City Hospital Campus, Nottingham University Hospitals NHS Trust, The Gables, Hucknall Road, Nottingham NG5 1PB, UK)
Recruiting Consultant Clinical Geneticists: Abhijit Dixit, Jacqueline Eason*, Ajoy Sarkar, Nora Shannon, Mohnish Suri
Research Nurse/ Genetic Counsellors: Ann Selby
Diagnostic Laboratory scientists: Gareth Cross, Katherine Martin

Oxford (Oxford Regional Genetics Service, Oxford Radcliffe Hospitals NHS Trust, The Churchill Old Road, Oxford, OX3 7LJ, UK)
Recruiting Consultant Clinical Geneticists: Edward Blair, Richard Gibbons, Usha Kini*, Sue Price, Debbie Shears, Helen Stewart
Research Nurse/ Genetic Counsellors: Julie Phipps**,** Abigail Pridham, Hellen Purnell
Diagnostic Laboratory scientists: Susan Clasper, Anneke Seller

Sheffield (Sheffield Regional Genetics Services, Sheffield Children's NHS Trust, Western Bank, Sheffield, S10 2TH, UK)
Recruiting Consultant Clinical Geneticists: Meena Balasubramanian, Diana Johnson, Michael Parker*
Research Nurse/ Genetic Counsellors: Louise Nevitt, Stuart Ingram, Cat Taylor
Diagnostic Laboratory scientists: Emma Shearing, Kath Smith

Southampton/Wessex (Wessex Clinical Genetics Service, University Hospital Southampton, Princess Anne Hospital, Coxford Road, Southampton, SO16 5YA, UK and Wessex Regional Genetics Laboratory, Salisbury NHS Foundation Trust, Salisbury District Hospital, Odstock Road, Salisbury, Wiltshire, SP2 8BJ, UK and Faculty of Medicine, University of Southampton)
Recruiting Consultant Clinical Geneticists: Munaza Ahmed, Diana Baralle, Amanda Collins, Nicola Foulds, Katherine Lachlan, I. Karen Temple*, Diana Wellesley
Research Nurse/ Genetic Counsellors: Lucy Harrison, Audrey Torokwa
Diagnostic Laboratory scientists: David J. Bunyan , Morag N. Collinson

Figures

Supplementary Figure S1. Detailed Outline of DDD Workflow

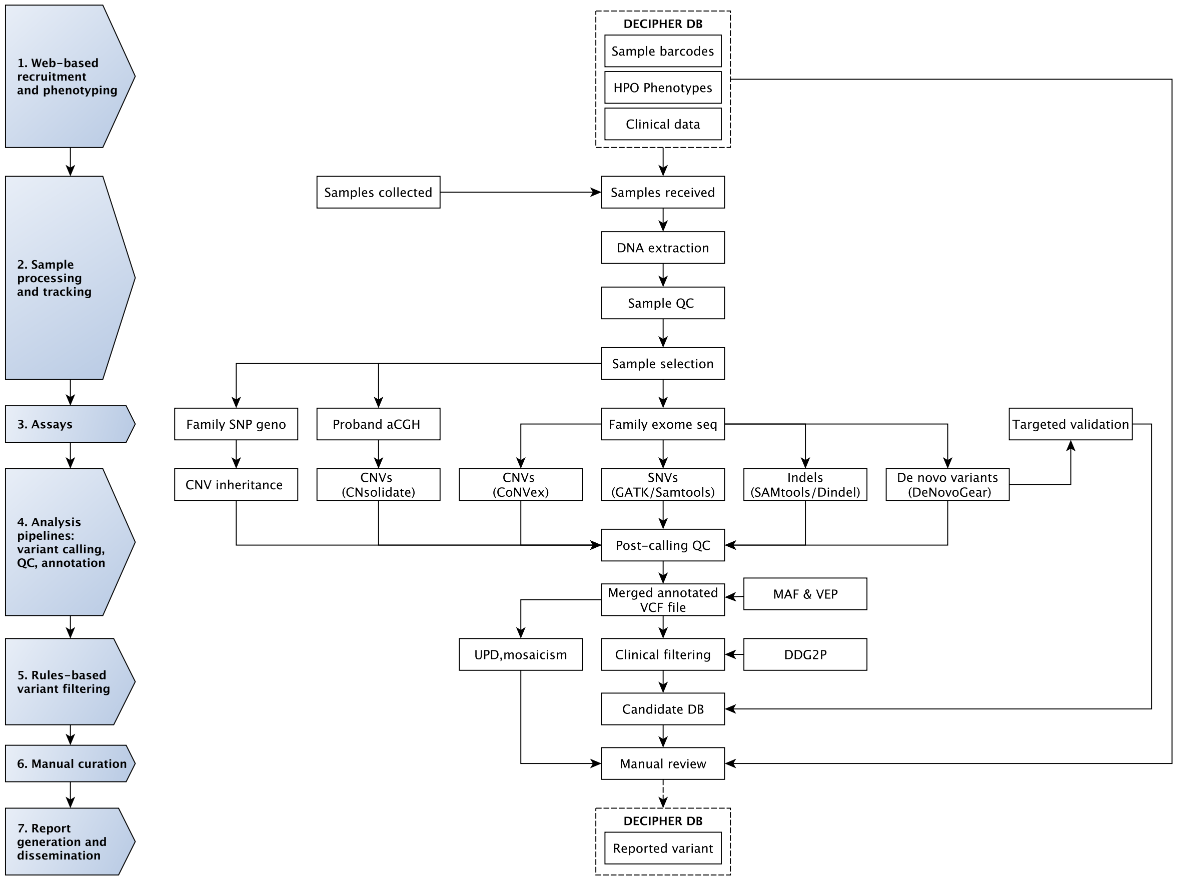

Supplementary Figure S2. Comparison of Variant Prioritisation Methods

ROC curves plotted using the pROC R-package[7] and distribution of scores comparing the performance of PolyPhen-2, SIFT, CADD, RVIS and MutationTaster for discriminating between reported and unreported rare missense variants in DDG2P genes, separated into *de novo* variants (which have a high prior probability of being causal) and inherited variants. AUC = area under the curve; CI = confidence interval.

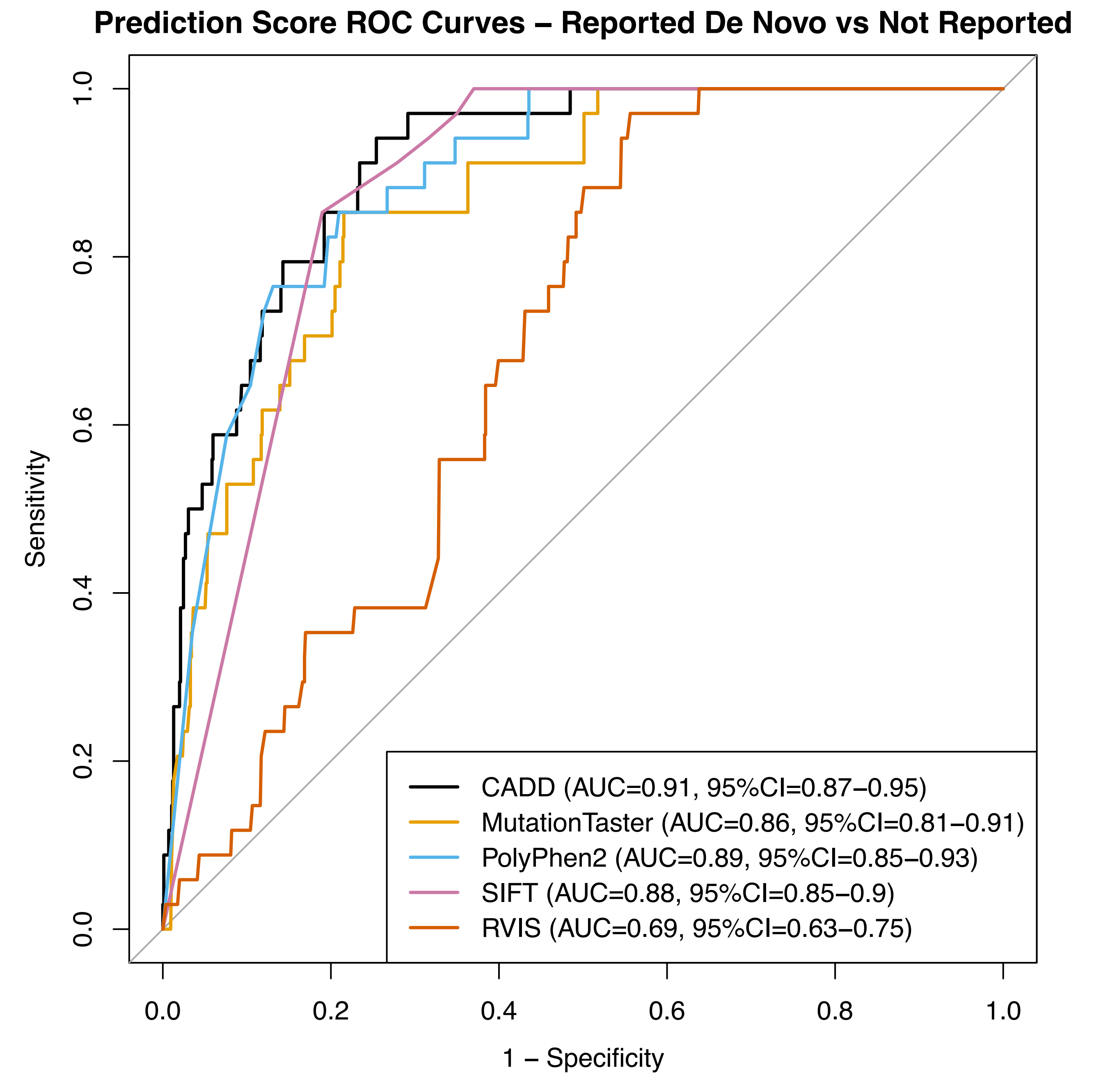

Prediction Score ROC Curves – Reported De Novo vs Not Reported
Sensitivity
1 – Specificity
0.0
0.2
0.4
0.6
0.8
1.0
CADD (AUC=0.91, 95%CI=0.87–0.95)
MutationTaster (AUC=0.86, 95%CI=0.81–0.91)
PolyPhen2 (AUC=0.89, 95%CI=0.85–0.93)
SIFT (AUC=0.88, 95%CI=0.85–0.9)
RVIS (AUC=0.69, 95%CI=0.63–0.75)

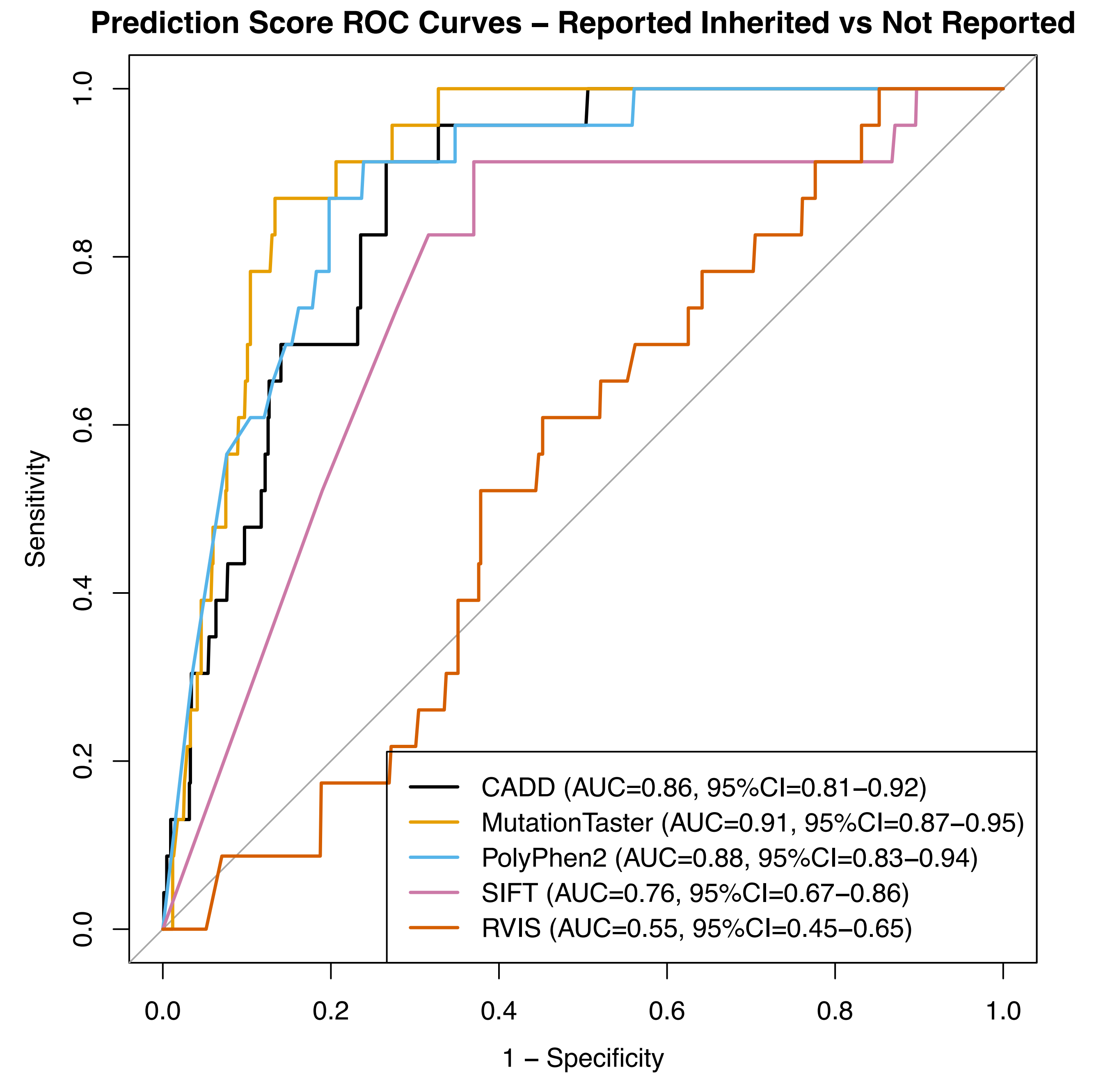

Prediction Score ROC Curves – Reported Inherited vs Not Reported
Sensitivity
1 – Specificity
0.0
0.2
0.4
0.6
0.8
1.0
CADD (AUC=0.86, 95%CI=0.81–0.92)
MutationTaster (AUC=0.91, 95%CI=0.87–0.95)
PolyPhen2 (AUC=0.88, 95%CI=0.83–0.94)
SIFT (AUC=0.76, 95%CI=0.67–0.86)
RVIS (AUC=0.55, 95%CI=0.45–0.65)

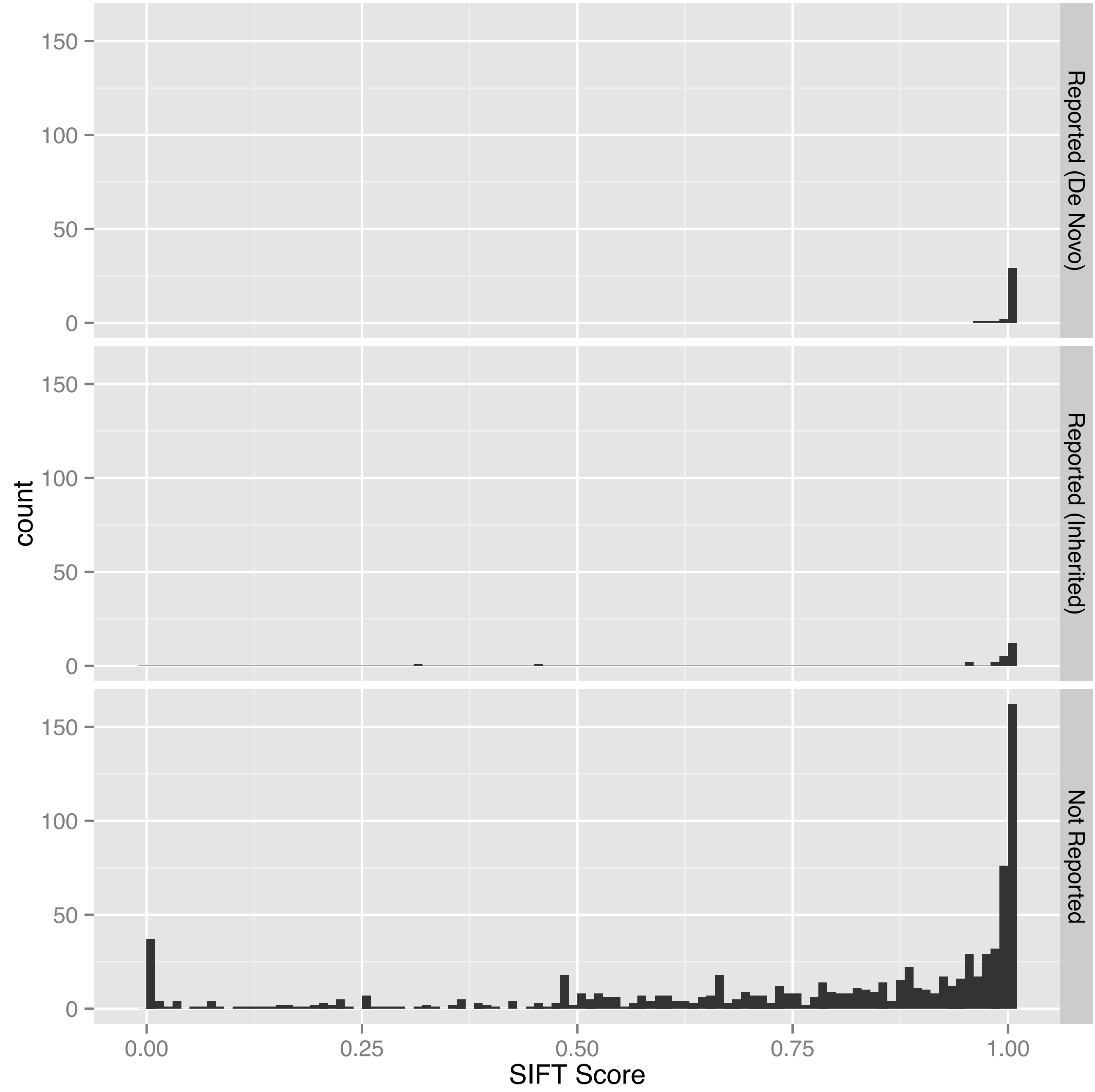

150
100
50
0
Reported (De Novo)
Reported (Inherited)
Not Reported
count
0.00
0.25
0.50
0.75
1.00
SIFT Score

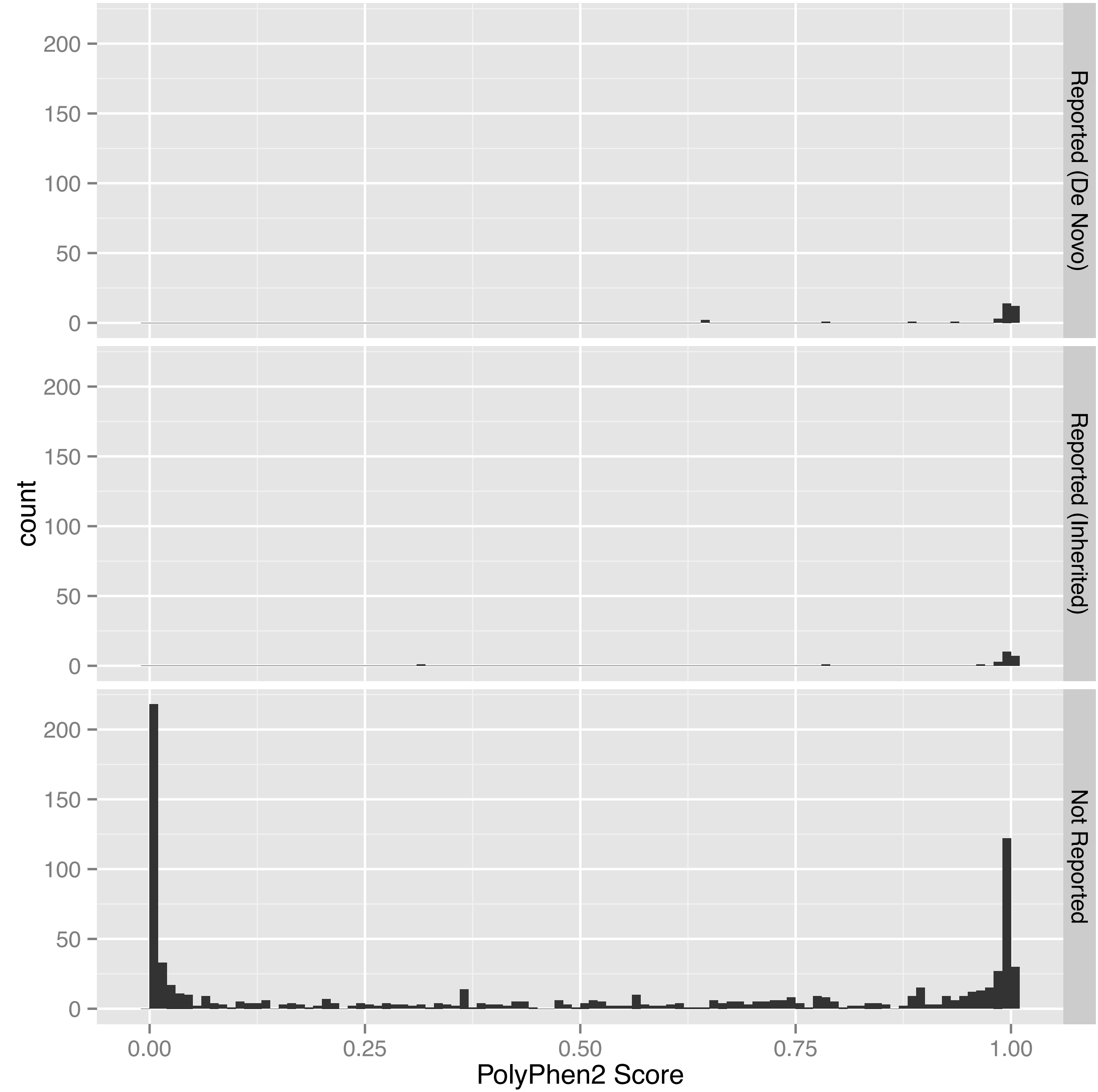
count
PolyPhen2 Score
Reported (De Novo)
Reported (Inherited)
Not Reported
0
50
100
150
200
0.00
0.25
0.50
0.75
1.00

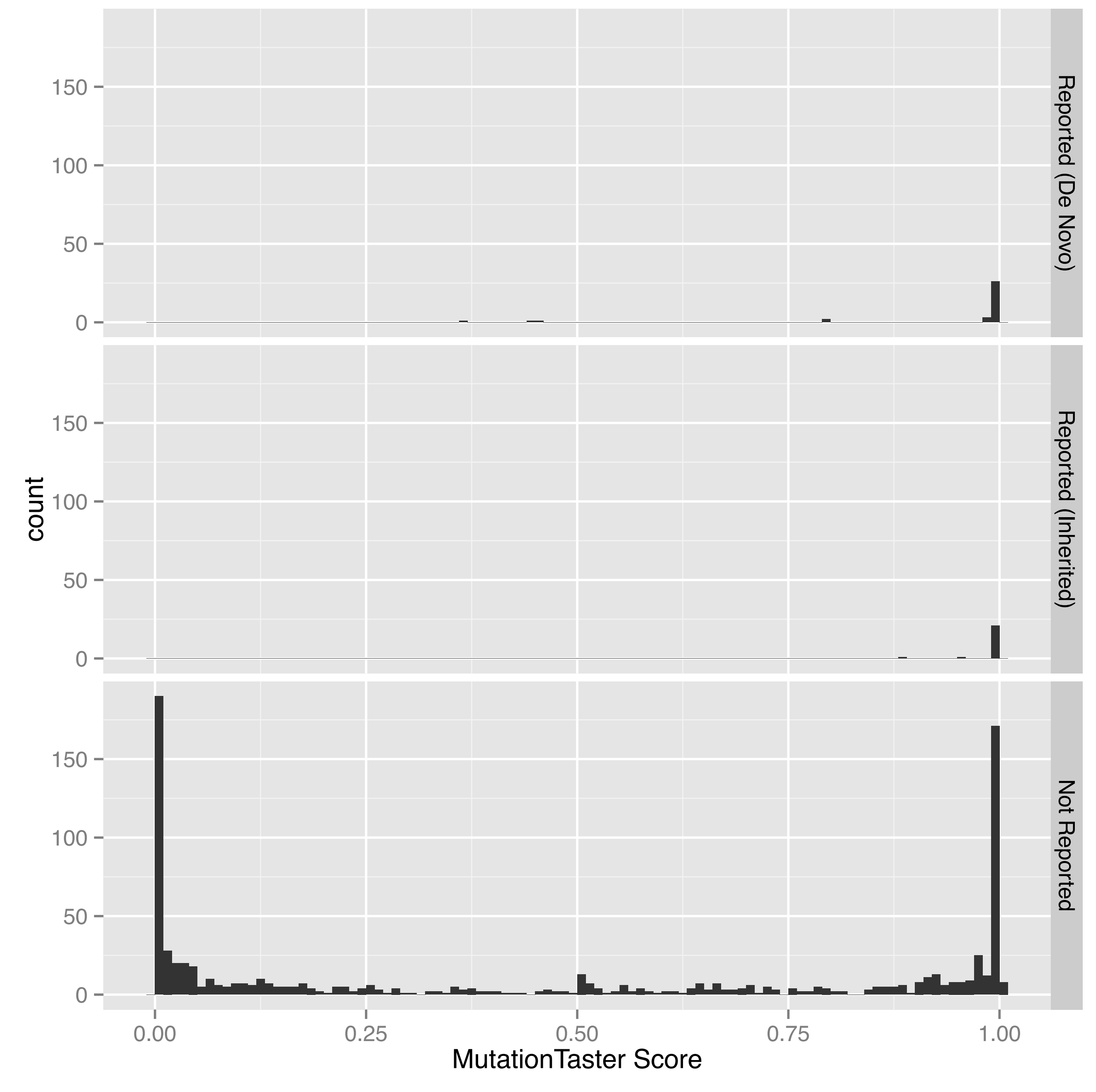
Reported (De Novo)
Reported (Inherited)
Not Reported
count
0
50
100
150
0.00
0.25
0.50
0.75
1.00
MutationTaster Score

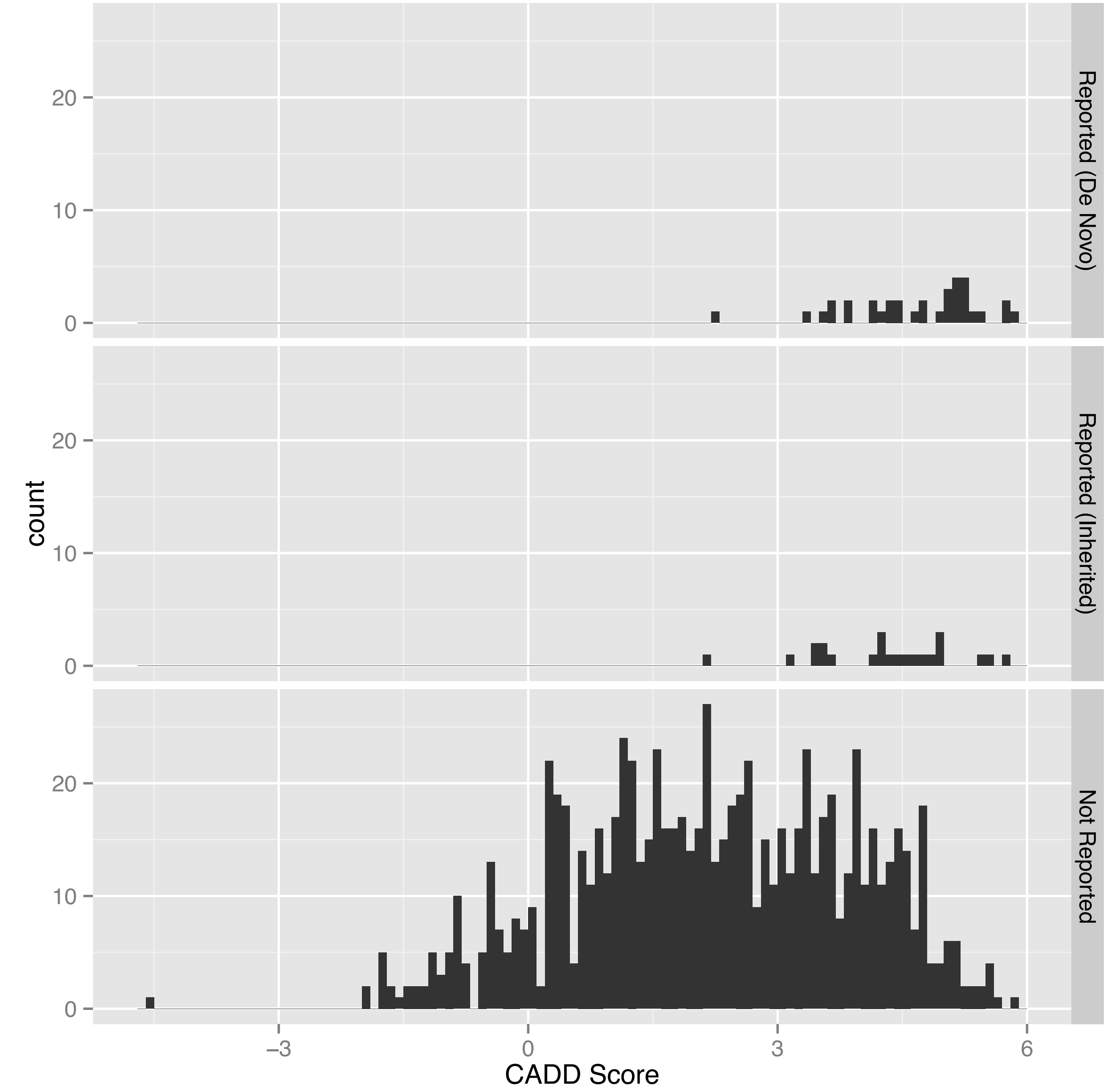

count
20
10
0
Reported (De Novo)
Reported (Inherited)
Not Reported
−3
0
3
6
CADD Score

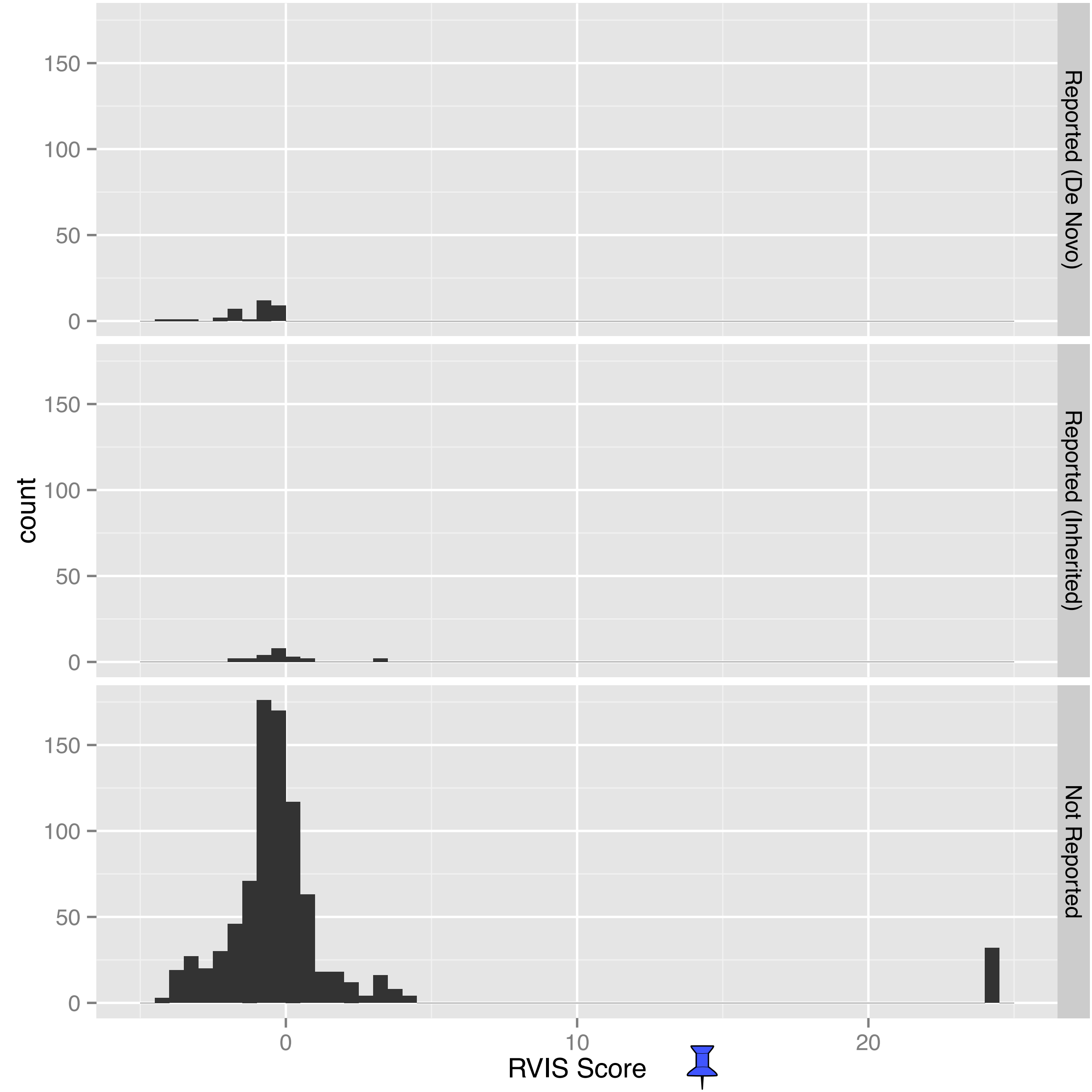

count
150
100
50
0
Reported (De Novo)
Reported (Inherited)
Not Reported
0
10
20
RVIS Score
